# The Use of Pepsine in the Diarrhœa of Infants

**Published:** 1868-08

**Authors:** Jas. S. Hawley

**Affiliations:** Greenpoint, L. I.


BUFFALO

### Medical and Surgical Journal.

VOL. VIII. AUGUST, 1868. No. 1.

### Original Communications.

ART. I.—*The use of Pepsine in the Diarrhœa of Infants.* By Jas. S. Hawley, M. D., *Greenpoint, L. I.*

In speaking of the cases of infantile diarrhœa, Dr. West, in his Treatise on the Diseases of Infants, makes the following remarks: "You will observe that the period of the greatest prevalence of diarrhœa coincides exactly with the time during which the *process of dentition* is going on most actively, and that more than half of all the cases of diarrhœa occur in children between the ages of six months and two years.

"The older writers on medicine, whose notice this fact did not escape, attributed the disturbance of the bowels to a sort of sympathy between the intestinal canal and the gums, swollen and irritated by the approach of the teeth to their surface. It must be borne in mind that there exists, during the period of teething, a *more abiding cause*, which strongly predisposes to its occurrence. All parts of the digestive canal, and of its dependencies, are undergoing an active evolution to fit them for the proper assimilation of the varied food on which the young being will soon have to subsist. Just as the salivary glands are now developed, and pour out saliva in abundance, so the whole glandular system of the intestines assumes a rapidity of growth and an activity of function, which, under the influence of comparatively slight exciting causes, may pass the just limits of health. In too many instances, causes fully adequate to excite diarrhœa are abundantly supplied in the excessive quantity or unsuitable quality of the food with which the infant is furnished; for it is forgotten that its condition is one of transition, in which something more than ordinary care is needed, while in accordance with that humoral pathology so popular among the vulgar, the profuse secretion from the irritated glands is regarded as the result of a kind of safety-valve arrangement whereby nature seeks to moderate the constitutional excitement attendant upon teething."

Here we have the *fons et origo* of infantile diarrhœa, an abiding, ever-present, predisposing cause, always ready to be quickened into activity by innumerable excitants. Errors of diet or depressing and painful emotions—as anger, fear, grief or anxiety; food, improper in quantity or quality; perturbing medicines and over-heating occupations on the part of the mother. The same errors of diet, changes of temperature and constitutional vices on the part of the infant, are ever ready and frequently occurring exciting causes.

This condition of transition which the teething infant exhibits is everywhere impressed upon animated nature. In the human family we have not only the great transition state of the alimentary and digestive systems at the period of dentition, in which we are now more particularly interested, but later the state of transition which attends the full development of the reproductive functions in which the nervous system is more particularly excited and liable to mal-development, and later still in life the second evolution in the generative system, when its functions, having fulfilled the order of nature, cease their activity. Again the mental functions and sentient system are in jeopardy. This danger to life and function which attends the great physical transitions in the human race finds its counterpart in the inferior creation. In insect tribes, the various transformations from egg to larva, from larva to chrysalis, from chrysalis to the winged insect, are each and all attended by dangers and destruction of life. Each shedding of the shell of the crustacea or skin of the reptile is a period of danger and frequently of death. Thus all nature speaks the same language and harmonizes with the teachings of science, that the periods of great change in the functions of the body are also periods of great danger. The proper functions of an organ or system of organs seem in whole or in part suspended or imperfectly performed during the period occupied by organic evolution. In other words, while the organ or system of organs, is being developed, or modified in structure, nature finds it necessary to rest from its labors, or materially diminish its activity.

In the normal condition of the human body the powers of nature are ample to carry on all necessary processes during the periods of evolution incident to the full development of the system. The vital machinery acquires sufficient momentum to carry its lagging and burdened wheels by the centres of obstruction which are met in each great developmental revolution. But in the artificial and morbid condition in which a large portion of infantile humanity is found, slight obstructions bring the machinery to a dead stand, or waste its powers in ineffectual attempts to continue its action.

This, to continue the figure, is the moment when the engineer by his lever may complete the revolution, and again all goes forward in harmonious action. This is the critical moment when a grain of sand or a drop of oil may increase or diminish the friction till on the one hand its motions may cease forever; or, on the other, proceed with its revolutions till its destiny is fulfilled.

Premising, then, that the great predisposing cause of infantile diarrhœa is the state of evolution which the digestive system and its dependencies are undergoing during the period of dentition, the question of therapeutics becomes one of comparative simplicity, and the evident duty of the physician is to allay that irritation of the organs which is exhibited in vomiting and purging, first, by the removal of all extraneous sources of disturbance, such as food, improper in quantity or quality, by protecting the skin from too sudden and frequent changes of temperature, secondly, by sedatives, to subdue the excitement which the foregoing causes may have induced, and which, in the enfeebled condition produced by the transition state, are self-propagating, and lastly, to impart to the struggling and overwhelmed digestive apparatus that assistance which will enable it to convert food from the character of a foreign, and therefore irritant, material, into nutriment which will reinvigorate the natural forces and enable them to accomplish successfully the great and necessary evolution through which they are passing. Happily the practice of administering excitants to the already over-stimulated glandular system, has nearly passed away. *Hydr. cretæ*, and *Hydr. chlor.*, are therapeutical formulæ seldom penned by one who opens his eyes to the light of physiological or rational therapeutics in infantile diarrhœa. As sedatives to the over-excited mucous membrane and glandular system of the stomach and bowels, the preparations of opium and the salts of bismuth stand preëminent. When irritation, without pain, exists, bismuth most promptly and satisfactorily allays it, but when accompanied with pain, the addition of a minute portion of opium becomes a necessary complement to its effectiveness.

We have now briefly noticed, in outline, the first two conditions of treatment, viz.: the removal of external causes of irritation, and the allaying of the morbid excitement which has sprung from their agency; and it may be asked if the natural functions will not now resume their offices, and the health of the patient be restored. Doubtless such would be the case did not the system labor under the combined effects of the transtition state of dentition and the impairment of strength due to the morbid causes above enumerated, and for which the correctives bave been proposed. But the circle of remedies is not yet complete. The key-stone of the arch is wanting, and if left thus incomplete, will tumble back into the disorder and confusion from which it has been raised. The *ingesta* themselves become, for want of digestive and assimilative power, irritants to the sensitive and debilitated organs. Instead of affording nutriment to fortify the system against the dangers of the crisis through which it is passing, the food going through the intestinal canal in an undigested form, becomes, itself, an irritant, and adds another morbid cause to those already existing. This is not all: the food does not always remain a simple, foreign substance, inducing irritation, but undergoes putrifactive decomposition, adding new and more active sources of disease.

Here, then, is the gap in which we are to stand. And what are the weapons we are to use? Tonics and stimulants, as indispensible as they often are, here become awkward and doubtful. They are indirect and secondary—whips to stimulate lagging energies and not power to perform the labor and lift the burden. Here the happy thought of Corvisart comes to our relief. The very function which is crippled we can replace; the very strength which is exhausted we can supply. By the administration of pepsine we at once convert the ingesta into nutriment. They not only cease to be irritants to the digestive organs, but are absorbed into the circulation, and become sources of power instead of weakness.

Now, we have fulfilled all the indications. First, to remove all sources of irritation from the quantity or quality of the ingesta, or change of temperature. Secondly, allaying irritation by sedatives. Thirdly, artificial digestion by the administration of pepsine. This simple but effective treatment is not new, but has more than once been presented to the profession for its approval.

In support of its efficacy, especially that portion which relates to artificial digestion, and which this paper is particularly intended to illustrate, a few cases will be brought forward. The first case is one reported in the *Revue Medico Chir. de Paris*, Dec. 1856.

M. X——, aged 4 years, was admitted into the Hospital of *St. Eugenie* on the 23d of November, 1854, under the care of M. Barthez. For many months this child had suffered from frequent diarrhœa until it was emaciated and debilitated to the last degree. The appetite was voracious and the stools contained much undigested food. In the first instance M. Barthez tried the effect of properly adjusted diet, with small doses of trisnitrate of bismuth, but without avail. He then tried the pepsine, giving a dose (grs. v) at the commencement of a meal composed of the ordinary food of the hospital. On the following day (the 1st of December, the stools were of a better color, and in other respects more natural than they had been before; encouraged with this result the same quantity of pepsine was ordered to be given before each meal.

Dec. 3d. No stool. This was the first day without a motion for many months.

Dec. 4th. Still no stool. The pepsine discontinued.

Dec. 5th. Two somewhat fluid motions, although there was no change in the diet. There was, however, no undigested matter in the motions. The child was much better in every respect.

Three weeks afterwards the child was discharged cured. M. Barthez, however, did not return to the pepsine, but contented him self with small doses of the trisnitrate of bismuth.

This case led M. Corvisart to try the effects of that remedy in the diarrhœa of very young infants.

2d Case. Alexander Lang, born on the 2d of August, 1855, was seized on the 25th of October, with diarrhœa, after a very obstinate attack of erythema and eczema. This diarrhœa was accompanied with frequent hiccough and vomiting. On the 3d o. November 8 grs. of pepsine were given night and morning. On the 4th the same treatment was continued; and now the vomiting and purging have disappeared, the stools have become natural, the child takes the breast with avidity. The pepsine discontinued.

Nov. 22d. The vomiting and purging have returned. M. Corvisart has again had recourse to the pepsine.

Nov. 23d. The vomiting and purging have ceased, and the stools are natural. From this time the little patient went on well.

M. Corvisart adds, that many cases of the kind have fallen under his notice, and that the acidified form of the pepsine, which he himself tried, was quite as efficacious in these cases as the neutral form proved to be in the hands of M. Barthez.

The writer has been in the habit of administering pepsine in the diarrhœa of fed and teething infants for several years with marked success.

Notes of former cases not having been preserved, a few which have occurred in the last few days must suffice.

July 19th. Thomas Kennedy, aged 15 months, has had diarrhœa a week, is fed, passages watery and contain undigested food.

℞ Am. Pulv. pepsine. Sub nit. bismuth, ää grs. 5 every 3 or 4 hours.

This single prescription terminated the disease.

July 20. John Kniester, aged 18 months, is teething, diarrhœa has existed ten days, passages very watery and frequent, and contain undigested milk.

℞ Am. Pepsine. Sub nit. Bismuth, ää grs. 5 every 4 hours.

This case was also relieved by a single prescription of ten powders.

August 2d. P. Quigley, aged 8 months, has had diarrhœa a week, and been treated by another physician by astringents and opiates without benefit.

℞ Am. Pepsine. Sub nit. bismuth ää ʒ in 10 powders, to be given every 3 or 4 hours.

This case also recovered completely under this single prescription.

August 5th. Mary Duryee, aged 11 months, has four incisors on each jaw, but no marked signs of the approach of other teeth.

Has suffered considerably from diarrhœa for two weeks. On the 5th the diarrhœa increased alarmingly, accompanied by vomiting and great prostration, as well as pain. The vomiting was allayed with liq. bismuth, and the following prescription made:

℞ Am. Pepsine. Sub nit. bismuth, ää ʒj. Pulv. opii, gr. i, divided into 12 powders and one given every 2 to 4 hours according to circumstances.

This treatment, with slight modifications, according to pain or frequency of the discharge, has been continued to the present time, (Aug. 10th,) with nearly complete relief to all the symptoms, and she is now out of danger.

August 5th. Robert Kelly, aged 9 months, is teething, has had protracted diarrhœa and vomiting, is much emaciated, and passes large quantities of undigested milk, which are highly offensive. He has been for some time under treatment before coming to me. Pepsine and bismuth were prescribed in the usual manner and continued three days, when the child died. It is to be noticed in this case that the coagulated milk disappeared from the stools on the second day, showing the efficiency of the pepsine.

The only remaining case to which I will allude is one rather of infantile inanition than of true diarrhœa.

July 28th. D. N——, an infant two weeks old, said to have been born in a plump and healthy condition. Its present state is one diametrically opposite. Its face is thin and skinny, exhibiting painfully the bony outline. It has thin, muddy, but not frequent alvine discharges, and vomits whatever it swallows, even to half a teaspoonful of its mother's milk. It lies stupid, with its eyes closed and refuses the breast. It has also an intense muguet. In this extremity I ordered three grains of pepsin to be given every three hours and half a teaspoonful of the mother's milk to be administered with great frequency. The following morning I found the mother, through utter hopelessness, had greatly neglected my directions. It was only through much persuasion and the coöperation of a friendly neighbor that she was induced to pursue the treatment. During that day the vomiting ceased, and on the following day the child took the breast and retained and digested its nourishment. From this day it steadily improved in condition, and its diarrhœa and muguet disappeared.

On the 8th inst., one week after my last visit, I was called to see its mother, and could hardly have recognized the infant which so lately had seemed in the last stage of inanition. Its face had acquired a comparative fullness, its color was restored, it nursed well and freely, and seemed as likely to live and thrive as any infant. This child was simply starving to death. What led to its condition of inanition I could not satisfactorily learn, but its state seemed most hopeless. This case illustrates, in a remarkable manner, how little assistance will restore the digestive faculty to its normal activity and enable it to perform its functions unassisted.

Without adding cases of a similar kind from our own experience, which would, perhaps, extend the list to a tedious length, we take pleasure in submitting the testimony of Dr. R. E. Van Gieson in favor of the efficacy of the preparations of pepsine in the diseases under consideration. The Doctor, in furnishing us with his notes, remarks: "I have found pepsine peculiarly fitted for the treatment of that fearful scourge of children, cholera infantum, *after* the more profound and violent initial symptoms have been subdued by direct sedatives, such as hydrocyaic acid, ice, creasote, and the like. It has seemed to me that when the vomiting and purging have by such measures been arrested, that the whole gastro and intestinal tract is utterly incapable of assimilating even the blandest articles of aliment. We stand as it were between the danger of starvation on the one hand and the peril of again irritating the intestines to the evacuation of exhausting discharges by the administration of food. Just here pepsine is *the* remedy. By its aid we can secure the digestion of food which would otherwise irritate. So long as the stomach is disposed to remain quiet we need not feel alarmed if for a day or two the discharges per rectum are somewhat frequent. An astringent and opiate suppository will control this, and in the meanwhile we are gradually bringing the intestinal tract to its normal condition, *i. e.* digestion.

"Another great advantage arising from the use of pepsine in this disease, has been rendered apparent by a careful comparison of cases treated by the most approved method in vogue some eight years ago. The medical gamut was then sedatives, opiates, astringents, tonics. It yielded good results, but the cases were a long time in getting well. There came a period when the pernitrate of iron and the salts of quinia seemed almost powerless. This might be the third week or the third month in the disease. There was no particular irritation of the stomach, but unless the astringent was given with great regularity and in augmented doses, the discharges still continued. In these protracted cases the gastro-intestinal system seems but a passive tube, through which the food passes pretty much as it entered the mouth, giving but little nourishment to the patient, and much annoyance to the atonized viscera. In these cases pepsine is very clearly indicated, and will slowly but quite certainly, aid in the digestion of judiciously selected nutriment, until the system can recuperate sufficiently to manufacture its own pepsine, when the artificial substitute can be withdrawn. The annexed case, illustrates the foregoing remark, in a very clear manner:

C. H., aged 13 months, hand fed, chiefly on condensed milk for last ten months; central and lateral incisors through; first came under my care July 29th. Past history: Has been under treatment by another physician for a week. Was taken in the beginning with vomiting and purging, which were in a measure subdued by the treatment. The child was then pronounced better and the visits discontinued.

Present condition: The child is much emaciated, face shrunken, and of that senile appearance, so indicative of the ravages of cholera infantum. The stomach is still irritable, and the bowels operate from 5 to 8 times in twenty-four hours. Discharges similar to chopped spinage. The child craves drink constantly.

℞ Bismuthi sub nit. pepsine, (American,) ää ʒi; make xii powders; S one every three hours; ice cold milk punch every three hours.

30th. Discharges diminished, but still fœtid. Thirst diminished, stomach less irritable, no vomiting during the night. Continue treatment with addition of ice cold mutton broth.

31st. Discharges diminished and less fœtid, no vomiting; withdrew the punch and substituted milk in barley water.

℞ pepsine, ʒi; make xii powders and give as before, with plenty of open air.

Under this treatment the child has steadily improved. The discharges are growing firmer, in consistency each day, and vary in frequency from one to three in twenty-four hours. The appetite being now fair, and the discharges nearly normal, the pepsine will be withdrawn, and port wine and tincture cinchona substituted.

Of course the writer does not intend to exalt pepsine to the position of a specific in infantile diarrhœa. It is only claimed that its use is one step in the right direction; that it is capable of removing one of the principal predisposing causes, to wit: the impairment of the digestive function by the evolution which occurs at the period of dentition, and of preventing the irritation which attends the passage and decomposition of undigested food.

A favorite observation of the writer is, to mark the gradual disappearance of undigested food and fetor from the evacuations, feeling assured that so much at least is well and in the right direction, whether the case proceeds favorably or not. Many other considerations influence the course of the disease, and these indications must be met by their proper remedies, according to the judgment of the practitioner.

I will close this hasty and imperfect paper by commending pepsine to a fair trial in the treatment of infantile diarrhœa, especially during the period of dentition. It has no noxious or perturbing qualities. It has physiological reasons in its favor, and has to some extent, borne the test of experience.

